# The Role of Epigenetic Mechanisms in the Pathogenesis of Hepatitis C Infection

**DOI:** 10.3390/biom14080986

**Published:** 2024-08-10

**Authors:** Justyna Żychowska, Maciej Ćmil, Patryk Skórka, Joanna Olejnik-Wojciechowska, Paulina Plewa, Estera Bakinowska, Kajetan Kiełbowski, Andrzej Pawlik

**Affiliations:** 1 Department of Physiology, Pomeranian Medical University, 70-111 Szczecin, Poland; justynazychowskaa@gmail.com (J.Ż.); cmilmaciej@gmail.com (M.Ć.); p.skorka04@gmail.com (P.S.); esterabakinowska@gmail.com (E.B.); kajetan.kielbowski@onet.pl (K.K.); 2 Independent Laboratory of Community Nursing, Pomeranian Medical University, 71-210 Szczecin, Poland; 3 Institute of Biology, University of Szczecin, 71-412 Szczecin, Poland; paulina.plewa@op.pl

**Keywords:** hepatitis C virus, HCV infection, hepatocellular carcinoma, epigenetics

## Abstract

Hepatitis C virus (HCV) is a hepatotropic virus that can be transmitted through unsafe medical procedures, such as injections, transfusions, and dental treatment. The infection may be self-limiting or manifest as a chronic form that induces liver fibrosis, cirrhosis, or progression into hepatocellular carcinoma (HCC). Epigenetic mechanisms are major regulators of gene expression. These mechanisms involve DNA methylation, histone modifications, and the activity of non-coding RNAs, which can enhance or suppress gene expression. Abnormal activity or the dysregulated expression of epigenetic molecules plays an important role in the pathogenesis of various pathological disorders, including inflammatory diseases and malignancies. In this review, we summarise the current evidence on epigenetic mechanisms involved in HCV infection and progression to HCC.

## 1. Introduction

Hepatitis C virus (HCV) is a global health concern with an estimated 1.5 million new infections in 2019 [1]. The virus can be transmitted through unsafe medical procedures, including injections, dental and surgical treatments, and haemodialysis, among others [1]. The acute phase of infection is mainly asymptomatic. In some populations, the disease can be self-limiting, but in others, a chronic form develops, which manifests as liver fibrosis, cirrhosis, progression to hepatocellular carcinoma (HCC), and hepatic decompensation [2]. The diagnosis is based on determining the levels of anti-HCV antibodies and HCV RNA. HCC is the most common primary liver cancer, and the number of deaths due to HCC is increasing [3,4]. This disease often develops due to HCV and hepatitis B virus (HBV) infections, alcoholism, or metabolic dysfunction-associated steatotic disease (MASLD). Understanding the complex mechanisms involved in the pathogenesis of HCV infection and HCC progression are crucial to introduce novel treatments in the future. In recent years, numerous studies have investigated the role of epigenetics in the pathogenesis of inflammatory and malignant diseases.

During transcription, only 3% of the human genome is transcribed into protein-coding messenger RNA (mRNA). The remainder is non-coding RNA (ncRNA), including microRNAs (miRNAs), long ncRNAs (lncRNAs), and circular RNAs (circRNAs), among others [5]. miRNAs are single-stranded molecules that regulate the expression of structural genes at the post-transcriptional level. Mature miRNAs consist of 19–25 nucleotides and mediate the post-transcriptional silencing of target genes by targeting the 3′-untranslated region (UTR) of mRNAs. However, there are exceptions in which miRNAs can stimulate and enhance mRNA translation [6].

lncRNAs have a linear structure, while circRNAs are ring shaped, but both classes share similarities. Specifically, both are composed of over 200 nucleotides and can be transcribed from exons, introns, and non-translated regions. Their unusual structures help them regulate gene expression via several mechanisms. They can influence transcription factors, enabling them to bind to promoters and thereby regulate specific gene expression. They function as scaffolds, controlling protein–protein interactions and related signalling pathways. They can also act as a decoy for miRNAs to prevent the degradation of target mRNAs. Recent studies have also shown that lncRNAs and circRNAs are involved in the epigenetic modulation of chromatin [5]. ncRNAs can be detected in the blood and other biological fluids of patients with cancer. Many of them have become prognostic indicators and diagnostic markers. Their connection with cancer involves acting as oncogenes and suppressors by regulating the initiation and progression of tumourigenesis. ncRNAs from viruses and host cells are also involved in interactions between viruses and host cells. ncRNAs from human immunodeficiency virus (HIV), cytomegalovirus, herpes simplex, Epstein–Barr virus (EBV), and HCV have been described. They are involved in modulating host gene expression, controlling host protein translation, inhibiting pro-apoptotic signals, and inhibiting the immune response. On the other hand, host-derived ncRNAs may have the opposite effect, inhibiting viral replication and enhancing the immune system response [7]. In this review, we discuss the current evidence regarding the involvement of epigenetic mechanisms in the pathogenesis of HCV infection and progression to HCC.

## 2. Epigenetic Mechanisms Regulating Hepatitis C Infection and Progression to Hepatocellular Carcinoma

### 2.1. DNA Methylation

DNA methylation is an epigenetic mechanism that is involved in regulating gene expression. Classically, DNA is methylated at CpG sites, which can create clusters (CpG islands). The main enzymes involved in the methylation process are DNA methyltransferases (DNMTs). While this modification can have several effects on gene expression, it is typically associated with gene repression. For example, methylation at the promoter region can suppress transcription [8]. However, methylation can also promote transcription by allowing the interaction with DNA-binding proteins [9]. Importantly, as DNA methylation affects gene expression, the identification of hypo- or hypermethylated regions might provide insights into the pathogenesis of particular diseases. Furthermore, large-scale studies that evaluate the methylome could be used in diagnoses or to estimate prognoses, as demonstrated in cancer studies [10]. In this section, we discuss whether DNA methylation is involved in HCV infection and HCV-related carcinogenesis and whether DNA methylation analyses can be used in diagnosis.

Zekri et al. analysed promoter methylation in patients with HCC infection undergoing antiviral treatment [11]. The authors found that *RASSF1A* gene methylation was associated with fibrosis, while monitoring the methylation of the *06MGMT* gene could identify potential responders to antiviral therapy. Another study demonstrated that RASSF1A promoter hypermethylation serves as a biomarker in HCC diagnostics [12]. It was reported that the RASSF1A gene is involved in anti-tumour processes. RASSF1A gene function impairment resulted in carcinogenesis [13]. It was indicated that the RASSF1A gene may be involved in the process of apoptosis, the stabilisation of microtubules, and DNA repair [14,15]. In patents with HCC, both inactivation and methylation are involved in the protection of infected cells via the inhibition of the Wnt and Hippo pathways [12,16]. In addition, the methylation status and subsequent alteration in gene expression might be implicated in the pathogenesis of HCV infection. In Huh7.5 cells, a population that permits infection, HCV stimulation was associated with elevated DNMT activity [17], thus suggesting altered methylation profiles. DNA methylation can be involved in an altered antiviral host response. Specifically, interferon (IFN)-α induces downstream signalling that activates antiviral response mechanisms. In an early study, researchers showed that the activation of the signal transducer and activator of transcription 1 (STAT1), an important IFN-α downstream element, is suppressed by the overexpression of HCV core proteins [18]. The suppression of STAT1 stimulated HCV replication [19]. Perhaps the virus can affect the methylation of STAT1 to alter antiviral responses, which are associated with tumourigenesis. It was indicated that macrophages with legumain knockout enhance tumour cells aging by the activation of the JAK1/STAT1 pathway [20]. In HCV-related HCC, there is markedly enhanced STAT1 methylation in patients [21]. DNA methylation and, consequently, the suppression of gene expression, could also mediate resistance to HCV infection. The expression of the transcription factor cAMP response element binding protein 3-like 1 (CREB3L1) is reduced in cells permissive for HCV [22]. Chen et al. [23] observed that the *CREB3L1* gene is methylated in cells permissive for the virus. Moreover, the authors made a similar observation regarding the myxovirus resistant 1 (*MX1*) gene, which is a known antiviral gene. *MX1* belongs to IFN-regulated genes, and its different methylation profile was also observed in patients with rheumatoid arthritis and Sjorgen’s syndrome [24,25], thus suggesting the potential role of *MX1* methylation in inflammatory responses as well.

Hypothetically, the methylation status of antiviral genes could be detected to identify individuals with an elevated risk of HCV infection. The virus also interacts with mechanisms that induce the degradation of viral core proteins. Specifically, E6AP enhances ubiquitin-dependent viral core protein degradation [26]. HCV has been suggested to induce the gene methylation of the *E6AP* gene, thus suppressing its expression and enhancing viral infection and replication. Mechanistically, the knockdown of DNMT1 could alter DNA methylation and thus reduce the expression of E6AP and HCV proteins [17].

### 2.2. Histone Modifications

In cells, DNA is organised into chromatin, which consists of nucleosomes as its basic units. Histones are key components of these nucleosomes, forming an octamer made up of the four core histone proteins (H2A, H2B, H3, and H4), around which a 147-base-pair segment of DNA is wound. Each histone protein has a unique side chain or tail, rich in basic lysine and arginine residues [27]. These histone tails undergo numerous covalent post-translational modifications (PTMs) that play a crucial role in regulating the chromatin structure. These modifications can change the charge density between histones and DNA, affecting chromatin organisation and the processes of transcription. Additionally, some PTMs act as signals for specific binding proteins that can alter the chromatin structure or function upon binding. Histone modifications encompass acetylation, methylation, ubiquitination citrullination, and phosphorylation [28]. Histone modifications impact cellular processes through two primary mechanisms. The first mechanism involves modifications that directly alter the chromatin structure, affecting it either locally or across larger regions. The second mechanism involves modifications that control the binding of effector molecules, either enhancing or inhibiting their attachment [29]. Histone acetylation and phosphorylation effectively reduce the positive charge of histones, which can disrupt electrostatic interactions between histones and DNA. This results in a less condensed chromatin structure, making DNA more accessible to protein complexes involved in processes such as transcription [30].

Viruses can manipulate the chromatin structure by altering its modifications, thereby affecting host–cell transcription and potentially leading to oncogenesis. In the context of HCV infection, disruptions in both DNA methylation and histone modifications have been documented [31,32]. Studies have shown that HCV infection significantly alters the genome-wide distribution of histone modifications, leading to extensive changes in the gene expression patterns in host cells. Remarkably, these epigenetic changes persist even after successful treatment with direct-acting antiviral (DDA) agents. This phenomenon has been observed in cell culture models, human liver chimeric mouse models, and human liver biopsies [33]. Research has shown that the overexpression of KDM5B/JARID1B, a member of the JmjC histone demethylase family, can increase HCC cell proliferation by regulating its downstream genes *E2F1* and *E2F2*, highlighting the role of histone methylation in HCV-induced HCC. Another histone lysine-specific demethylase, LSD1, also linked to HCV infection in HCC, is elevated in HCC cells compared with normal cells. Cells lacking LSD1 exhibit higher levels of H3K4me1/2 and H3K9me1/2 [34,35].

Histone acetylation, which is dynamically regulated by histone acetyltransferases (HATs) and histone deacetylases (HDACs), plays a significant role in the development of HCV-infected HCC. This includes modifications such as the acetylation of histone H3 at lysine 9 (H3K9Ac), histone H3 at lysine 27, histone H2A at lysine 5, and histone H3 at lysine 14. In mice, knocking out HDAC3 increases H3K9Ac and decreases the trimethylation of histone H3 at lysine 9 trimethylation in mice. This alteration disrupts the repair of double-strand breaks (DSBs) and encourages the development of HCC [36]. H3K27Ac, primarily found in the central euchromatin regions of cancer cells, activates global cell-type-specific gene expression. HCC tissues exhibit higher levels of acetylation of histone H3 at lysine 27 compared with normal liver tissue regardless of whether the infection is caused by HCV or HBV [37]. Histone acetylation related to HCC involves multiple biological processes. For example, iron overload, a risk factor for HCV progression to HCC, is normally regulated by human hepcidin, a peptide produced by the body. In the case of HCV infection, hepcidin expression is decreased due to HDAC activation, affecting histone acetylation rather than DNA methylation [38]. Hepcidin potentially defends against HCV by inhibiting its replication through the activation of the STAT3 signalling pathway [39,40]. It has also been reported that vasohibin 2 (VASH2) is abnormally overexpressed, promoting HCC proliferation and inhibiting apoptosis. This overexpression is associated with the decreased trimethylation of histone H3 at lysine 27 and the increased trimethylation of histone H3 at lysine 4 and histone H3 acetylation in VASH2 promoters [41].

**Figure 1:**
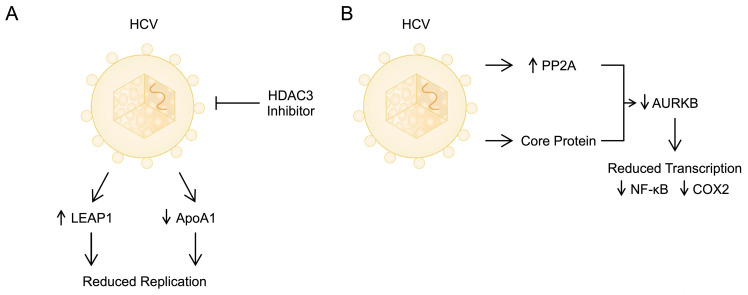
(**A**) The histone deacetylase 3 inhibitor modulates the impact of HCV on LEAP1 and ApoA1, which consequently suppresses viral replication [42]. (**B**) HCV affects the expression of PP2A to inhibit the activity of AURKB, thus reducing the expression of pro-inflammatory mediators and enhancing the immunosuppressive environment. LEAP1—liver-expressed antimicrobial peptide; ApoA1—apolipoprotein A1; PP2A—protein phosphatase 2A; AURKB—aurora kinase B; NF-κB—nuclear factor kappa-light-chain-enhancer of activated B cells; COX2—cyclooxygenase 2.

HDACs have been positively associated with HCC and HCV. An HDAC3 inhibitor reduced HCV replication by increasing the expression of liver-expressed antimicrobial peptide (LEAP1) and decreasing apolipoprotein A1 (ApoA1). Consequently, it has been evaluated for use in the clinical treatment of HCC. The antiviral action of this HDAC3 inhibitor may be due to transcription factors like hypoxia-inducible factor 1 (HIF1) and STAT3 altering histone acetylation in the promoter regions of these genes [42]. Lipoproteins are significantly associated with HCV infection. Specifically, through interactions with apolipoproteins, they form infectious apo-lipo particles [43]. As ApoA-I has been found to participate in viral replication [44], the effect of HDAC3 might represent another beneficial mechanism of action (Figure 1). Histone lysine or arginine methylases are closely linked to the prognosis of HCC. G9a, a histone methyltransferase, primarily drives the epigenetic silencing of the tumour suppressor gene *RARRES3*, aiding in the development of HCC [45].

HCV proteins can disrupt the activities of histone-modifying enzymes, which are essential for regulating gene expression and are connected to the onset of HCC. For example, the HCV core protein hampers histone acetylation by inhibiting HATs such as p300 and CBP. This interference decreases histone acetylation levels, suppressing the transcription of genes necessary for the antiviral defence, thereby promoting viral persistence and contributing to cancer development. The changes in epigenetics caused by HCV proteins help the virus persist and foster an environment favourable for cancer. By modifying histones, HCV evades the immune system and establishes chronic infection, which can lead to HCC. HCV proteins also impact HDACs. Specifically, the viral non-structural protein NS5A interacts with and inhibits HDACs, leading to changes in histone deacetylation patterns and disrupting the regulation of host gene expression. This disruption increases the expression of genes that support viral replication and survival, while decreasing the expression of genes involved in the host’s immune response [46,47,48].

HCV infection triggers an increased expression of protein phosphatase 2 catalytic subunit alpha (PP2CA) via an endoplasmic reticulum stress response pathway. Additionally, it has been observed that PP2A can interact with protein arginine methyltransferase 1 (PRMT1) and inhibit its enzymatic function. PRMT1 plays a crucial role in various biological processes, including signal transduction, protein localisation, gene transcription, and DNA damage repair. Notably, PRMT1 is also essential for epigenetic modifications of histones that regulate the chromatin structure and gene transcription. For example, PRMT1 facilitates the methylation of histone H4 at arginine 3. HCV infection markedly increases the expression of PP2A, a phosphatase that removes phosphate groups from Aurora B kinase (AURKB), thereby suppressing its kinase activity. Consequently, HCV core protein could potentially deactivate AURKB by activating PP2A [49,50].

Research has demonstrated that elevated PP2A expression led to significant alterations in the PTMs of histones, resulting in changes to the expression of genes associated with tumour development. Moreover, increased PP2A expression hinders DNA damage repair. These discoveries outline a pathway for tumourigenesis that connects HCV infection with the modified expression of oncogenes and compromised DNA damage response [50]. Another study showed that HCV and its core protein suppress the phosphorylation of histone H3 at serine 10. This inhibition occurs because the HCV core protein directly interacts with AURKB, leading to a reduction in AURKB activity. HCV and its core protein significantly decrease the transcription of NF-κB and cyclooxygenase 2 (COX-2), two proteins known for their anti-apoptotic and proliferative effects in regulating the inflammatory response. The depletion of AURKB attenuates the repression of NF-κB and COX-2 gene transcription by HCV and its core protein, while the overexpression of AURKB reverses the viral effect. The inhibition of AURKB increases the specific infectivity of HCV, whereas the overexpression of AURKB reduces it [51]. Cancer cells are characterised by aneuploidy; therefore, alterations in processes such as the spindle checkpoint, chromosome segregation, or cytoknesis, which are regulated by Aurora B, may contribute to carcinogenesis [52]. Interestingly, Zhang et al. noted a higher expression of AURKB in tissues derived from HCC. Additionally, AURKB knockout induces the arrest of proliferative cells in the G2/M phase. AURKB can be detected in serum, which may become a promising prognostic biomarker and predictor of HCC prognosis in the future [53]. In summary, numerous studies have demonstrated that viral oncoproteins interact with and stimulate the expression of cellular DNMTs and various histone-modifying enzymes such as HDACs, HATs, histone methyltransferases, and demethylases, thereby altering their function [54].

### 2.3. Non-Coding RNAs

#### 2.3.1. MicroRNA

Numerous studies have increasingly focused on the role of miRNAs in the pathogenesis of HCV. Their significance as biomarkers of disease severity and inflammation-mediated fibrosis has been emphasised. Dysregulated miRNAs play an important role in modulating HCV infection, as they can both stimulate and inhibit infection [55]. HCV entry into hepatocytes is blocked by miR-548m and miR-194, which inhibit the expression of CD81; miR-182, which suppresses the expression of CLDN1; and miR-122 and miR-200c, which suppress the expression of OCLN. At the level of replication inhibition, miR-196, miR-296, miR-351, miR-431, and miR-448 interact directly with the HCV genome; miR-199a and let-7b interact directly with the 5′-UTR region of the HCV genome; and miR-181c interacts directly with the regions encoding the E1 and NS5A proteins of the HCV genome. On the other hand, miR-122 facilitates the replication of the HCV genome by directly interacting with the 5′-UTR. miR-99a suppresses HCV assembly, while miR-501-3p and miR-619-3p facilitate it [56].

The most studied miRNA is miR-122, which accounts for a significant proportion of all miRNAs in the liver [57]. miR-122 has an unconventional role, as its elevated expression leads to increased translation and stabilisation of HCV RNA. The association between HCV and miR-122, which is thought to be essential for viral replication, results from the binding of the 5′-UTR of HCV to two miR-122 molecules. The 5′-UTR region contains the internal ribosome entry sequence (IRES), which regulates the translation of the viral polyprotein. This polyprotein, 3000 amino acids long, is proteolytically processed by host and virus proteases into 10 individual viral proteins [58,59]. In addition, the stimulation of the HCV IRES by miR-122 is essential for efficient viral replication through alternative folds involving the miR-binding region (MBR) in a manner similar to RNA chaperone proteins. Energetically favourable alternative secondary structures adjacent to the IRES are inhibited, disrupting its function [60]. The expression of miR-122 in the liver is relatively constant and integrated into the circadian clock output system, with the orphan receptor REV-ERBα being the dominant regulator of circadian mir-122 transcription. In addition, the downregulation of miR-122 has been shown to dysregulate multiple mRNAs that accumulate in a circadian manner [61]. In patients with fibrosis who are infected with HCV, the liver miR-122 levels decrease significantly with the progression of fibrosis severity [62]. The role of miR-122 is not limited to HCV infection; there are many links to HCC in which reduced levels of miR-122 are found [63,64,65]. Decreased miR-122 levels may be associated with a poor prognosis [64], including liver cancer metastasis [65]. Miravirsen is a drug that targets miRNAs and has the potential to inhibit miR-122. This oligonucleotide (15 base pairs) has been tested in a phase 2 clinical trial with good results. It exerted antiviral activity for all HCV genotypes in a chimpanzee model infected with HCV, with no evidence of viral resistance [66,67]. An in vitro study demonstrated that miravirsen exhibits additive activity in combination with NS3, NS5B, and NS5A inhibitors and remains fully active against HCV replicons resistant to these inhibitors. The broad antiviral activity of miravirsen against both wild-type and DAA-resistant HCV replicons, combined with its relatively high barrier to resistance due to interactions with host functions, underscore the advantages of miR-122 as a therapeutic target [68].

miR-155 plays an important role in the stimulation of monocytes and natural killer (NK) cells associated with the inflammatory process. In addition, it is a target for several inflammatory mediators. Mediated by T cell growth, miR-155 is also responsible for inducing autoimmune inflammation [69]. It has also been shown to play an important role in the human body’s response to HCV infection. Increased miR-155 expression has been observed in serum and peripheral mononuclear blood cells (PBMCs) in patients with long-term HCV infection. It is likely that this condition reflects progressive liver damage associated with inflammation [70]. After treatment, there is a decrease in the amount of miR-155, which may suggest that increased expression promotes HCV infection [71]. In the context of HCV infection, miR-155 affects the replication and life cycle of the virus. In addition, it affects the innate immune response associated with promoting NK cells to release IFN [72]. miRNA deficiency is associated with the inhibition of the cell cycle of hepatocytes in the G0/G1 phase. On the other hand, the overexpression of miR-155 contributes to inhibit apoptosis in liver cells and affects their proliferation and carcinogenesis as a result of increased Wnt signalling [71]. It is likely that miR-155 affects the development of a tumour in the direction of HCC by suppressing tumour suppressor genes [72]. Interestingly, HCV itself increases miR-155 expression both in vivo and in vitro by NF-κB [71]. In addition, it is possible to trigger miR-155 secretion via TLRs closely associated with HCV [73]. miR-155 has diagnostic potential; in particular, miR-155-5p may have considerable value in the prognosis of HCC. Its significant expression has been observed in HCC tissues and is closely related to TNM classification [74].

miR-223 affects non-specific mechanisms related to the differentiation of monocytes, neutrophils, and granulocytes. There are elevated miR-223 plasma levels during HCV infection, manifesting a long-term immune response. The effect of increased miR-223 plasma levels on liver cells is unclear [75]. However, miR-223 expression in liver cells has been shown to be reduced due to chronic HCV infection [76]. In addition, miR-223 modulates the insulin-like growth factor receptor, whose elevated expression is associated with the level of malignancy of a liver tumour [77]. miR-223-3p is correlated with HCC as a consequence of HCV infection. It is associated with NOD-like receptors (NLRs), which play an important function in HCC biology through NLRP3 by aggravating apoptosis and hepatocyte fibrosis [78]. However, miR-223-3p can only serve as a marker of liver damage because it is not specific to either HCV or HCC [79]. Interestingly, researchers have reported increased miR223-2p expression for several drugs, including DAA and ombitasvir/paritaprevir/ritonavir + dasabuvir ± ribavirin [80].

miR-29, including miR-29a, miR-29b, and miR-29c, acts on extracellular matrix (ECM) proteins such as collagen, elastin, fibrillin, and laminin [81]. During HCV infection, the miR-29 serum levels increase, while the liver levels decrease compared with those in healthy people [82,83]. Studies have shown that overexpression has a positive effect on the suppression of HCV replication, indicating that it is an antiviral defence element. In addition, it may be a potential therapeutic agent in anti-HCV treatment by regulating inflammation and fibrosis [82]. Interestingly, miR-29 liver levels are also increased in people diagnosed with chronic HCV infection with a long-term virological response, as opposed to patients without a long-term virological response [84]. In light of this research, it can be concluded that miR-29 is responsible for the surveillance of HCV infection. However, HCV can modulate miR-29 expression, although the mechanisms are not yet fully understood. It is postulated that this can be achieved through the stimulation of transforming growth factor (TGF)-β [85]. Pedersen et al. [86] revealed that the use of IFN stimulates the expression of several miRNAs, including miR-29; this approach could be used to treat HCC. In tumours, miR-29 plays a suppressive role by regulating processes such as apoptosis, proliferation, angiogenesis, and fibrosis [87]. miR-29 overexpression slows down tumourigenesis via the anti-apoptotic proteins BCL2 and MCL1. It also contributes to the decrease in HCC cell migration but stimulates the migration of CD8+ T cells [87,88].

miR-34 also plays an important role in HCV infection: its expression is particularly increased in serum [89]. miR-34 expression is also correlated with the HCV-dependent form of cirrhosis. People with severe fibrosis have increased levels compared with people with mild or moderate disease [90]. The role of miR-34 is associated with the regulation of the cell cycle [91]. An interesting finding is that HCV increases the expression of miR-34 in target cells and thus blocks further viral replication. This is probably due to IFN, which is associated with the inhibition of replication in other flaviviruses, such as dengue virus or West Nile virus [91]. In addition, an increase in miRNA-34 expression is associated with p53 stimulation and the release of miRNA-containing extracellular vesicles after HCV infection. miR-34 released in this way suppresses HCC [89,92]. In cancer cells, miR-34 inhibits the cell cycle and stops apoptosis-related mechanisms [92]. Therefore, miR-34 is a good marker of HCV-related HCC [93]. Table 1 summarises the miRNA molecules involved in HCV infection and progression to HCC.

#### 2.3.2. Long Non-Coding RNA

The concentrations of lncRNAs may increase after HCV infection. The increase in some lncRNAs enhances the body’s antiviral response by increasing the expression of interferon-stimulated genes (ISGs) or by directly blocking viral replication. On the contrary, some lncRNAs enhance the proviral response to increase virus viability [94]. For example, the lncRNA growth arrest-specific transcript 5 (GAS5) plays an important antiviral role in HCV infection by binding the viral NS3 protein, which belongs to the replicase complex [95]. Some lncRNAs can modulate the body’s immune responses by inducing or limiting the strength and duration of the response to IFN. HCV can evolve to force the cell to express lncRNAs that act as negative regulators of IFN, weakening the cellular antiviral response. Specifically, Zeng et al. [96] demonstrated that the lncRNA urothelial carcinoma-associated 1 (UCA1) affects antiviral response by downregulating the expression of IFN. Mechanistically, UCA1 can act as a sponge for miR-145-5p, which affects SOCS7. A lower expression of IFN indicates a poorer antiviral response. Another lncRNA that reduces the expression of ISGs and, consequently, the level of IFN, is EGOT, the transcription of which is induced by NF-κB activated by PKR after HCV infection [97]. It has been reported that similar mechanisms for regulating the response to IFN have also been observed in other viruses. These include Japanese encephalitis virus, dengue virus, group C enteroviruses, adenoviruses, and Kaposi’s-sarcoma-associated herpes virus [98]. New research on lncRNAs that modulate immune responses is necessary because they may open the way to the development of innovative treatments for infectious diseases.

The lncRNA HOTAIR contributes to efficient virus shedding by altering the lipid metabolism. Other lncRNAs, such as lncRNAs insulin-like growth factor 2 antisense RNA (IGF-AS) and the RNA component of 7SK nuclear ribonucleoprotein (7SK), increase the level of phosphatidylinositol 4-phosphate (PI4P) and, therefore, also participate in promoting virus release [95]. PI4P is a product of phosphatidylinositol 4-kinase and, together with its effector, oxysterol binding protein, is involved in maintaining the integrity of the HCV replication membrane [99]. lncRNA-IFI6 modifies histones responsible for transcription through their methylation, thereby stimulating HCV replication [100]. The concentration of another lncRNA, Linc-Pint—responsible for inhibiting de novo lipogenesis by binding to serine-arginine protein kinase 2 (SRPK2)—is reduced in HCV-infected liver cells [101].

Several lncRNAs increase after HCV infection and have oncogenic effects, which may contribute to the occurrence of HCC, among other neoplasms. The lncRNA NEAT1 is associated with several malignancies. HCV infection induces the increased expression of NEAT1, which leads to a decrease in the level of miR-9-5p and, ultimately, to an increase in the level of BGH3, an oncogene associated with HCC progression. Interestingly, patients treated with antiviral drugs also have higher NEAT1 concentrations, indicating that HCC may be induced by NEAT1 [102]. Interactions between the viral genome and cellular RNA should be explored in greater depth in the future. There are reports that viral lncRNAs with an as yet unknown function may be generated due to the processing of the end of the viral 5′ IRES sequence by the cellular endoribonuclease exoribonuclease 1 (XRN1), which inhibits the translation of viral proteins [95]. Interestingly, researchers found that the repression of an mRNA decoy in HCV-infected liver cells was associated with the repression of XRN1. The destabilisation of mRNAs, including those encoding oncogenes and immune regulators, sheds new light on the impact on the non-coding regions of the 5′-UTR of HCV in the molecular pathogenesis of HCC [103]. Liver cancer cell self-renewal through cancer stem cells (CSCs) is associated with the increased expression of lncTCF7, which regulates the activity of the Wnt signalling pathway, implicated in the progression of HCC. Mechanistically, lncTCF7 can act as a sponge for the SWI/SNF complex, which affects TCF7. Consequently, a higher expression of TCF7 indicates the self-renewal of liver CSCs. However, CSCs contribute to the aggressiveness of HCC through high recurrence rates and heterogeneity [104].

The overexpression of the antisense lncRNA LINC00624 disrupts the formation of the HDAC6-TRIM28-ZNF354C transcriptional corepressor complex, which moves it away from the specific CHD1L and BCL9 promoter loci, thereby removing the inhibitory effect on transcription in HCC. Thanks to this discovery, LINC00624 could be used as a therapeutic target for the treatment of HCC [105].

HULC is highly expressed in HCC, so it can be used as a tool for early HCC diagnosis [106]. Liver carcinogenesis is increased by HULC because it stimulates cyclin D1 and inhibits P21 WAF1/CIP1 via the autophagy-miR-675-PKM2 pathway in human liver CSCs [107]. Gaber et al. [106] reported increased HULC expression in patients with HCC as well as in a group of patients infected with HCV; however, HULC was significantly higher in the HCC group compared with the HCV group. Interestingly, HULC manipulates the pool of lipids that play a key role in the HCV life cycle. By reducing the interactions between lipid droplets and the HCV core protein, HULC facilitates the release of virus particles. Additionally, there are reports that the HCV core protein itself can regulate HULC transcription via retinoid X receptor alpha (RXRA) through changes in DNA methylation marks on HULC promoters. It will therefore be interesting to investigate these methylation mark changes in more depth to better understand the impact of HCV on HULC expression [108].

The concentration of another lncRNA, LINC01189, is significantly reduced in patients with HCC, especially those infected by HCV. Moreover, it has been mechanistically proven that LINC01189 can bind to and inhibit hsa-miR-155-5p, thereby reducing chemoresistance to 5-FU and the proliferation of HCC cells. Additional research on strengthening the LINC01189/hsa-miR-155-5p mechanism may prove groundbreaking in the treatment of HCC [109].

There are also reports of lncRNAs with anticancer potential. One of them is the lncRNA MEG3, the concentration of which decreases in the presence of HCC. Researchers showed that upon the expression of MEG3 with or without the HCV core protein (C191), there was significant upregulation of the tumour suppressors miR-152 and p53, as well as downregulation of the oncogenes TGF-β, BCL-2, and DNMT1. Additionally, there was a decrease in the tumour marker MKI67 and an increase in the apoptotic marker caspase-3. Another core protein (C173), together with MEG3, induced apoptosis in an HCC cell line [110]. Another study elucidated MEG3 downstream targets, including miRNA-10a-5p and PTEN, which inhibit cancer cell proliferation through PTEN/AKT/MMP-2/9 signalling [111]. Gana et al. [112] demonstrated that the lncRNA CASC2 affects the induction of apoptosis and the inhibition of HCC proliferation, invasion, and migration by inactivating the mitogen-activated protein kinase (MAPK) signalling pathway. The authors also speculated that future research on the detailed mechanism of action of CASC2 on the MAPK signalling pathway may contribute to the development of therapy for patients with HCC. The role of CASC2 with HCC was confirmed by a study in which significantly reduced CASC2 was observed in patients with HCV-related HCC compared with those infected with HCV alone [113].

The progression of HCV-induced chronic hepatitis C (CHC) into HCC is difficult to detect because atypical changes may initially be discreet. There are reports that the lncRNA HEIH derived from HCC cell lines and tissues may become a diagnostic target for this cancer. Zhang et al. [114] discovered that an increase in HEIH serum levels may also become a diagnostic target for HCC. Patients with HCV-related HCC presented an increased expression of HEIH in the serum and exosomes, but the ratio of HEIH levels in serum to exosomes was lower than that in patients with CHC. The authors hypothesised that exosomes, as structures that can store and release genes, release lncRNAs into the serum so that they can further regulate the expression of genes, including those involved in metastasis. HEIH may also be a useful biomarker in detecting and diagnosing liver cirrhosis and gastric cancer, among other conditions [115,116].

Another lncRNA, TUG1, is significantly upregulated in patients with both HCC and HCV compared with those with HCV alone. TUG1 is a known oncogene whose concentration is also increased in other cancers, including osteosarcoma, urothelial carcinoma of the bladder, gastric cancer, and oesophageal squamous cell carcinoma. Additionally, researchers found that the higher the TUG1 concentration, the higher the AFP concentration, and an increase in AFP is associated with a larger tumour volume and a lower median survival rate for patients with HCC [113,117].

The lncRNA MALAT1 [118] is significantly upregulated in patients with HCV-related HCC compared with those infected with HCV only. The combination of MALAT1 diagnostics with AFP may become useful in the diagnosis, prognosis, and screening of HCC. MALAT1 may also be a biomarker for diagnosing and detecting other cancers, such as breast or lung cancer [119]. Additionally, LINC01419 is significantly increased in both HCV- and HBV-dependent HCC. TO note, LINC01419 regulates the cell cycle and promotes tumour growth [120]. In summary, lncRNAs play a role in cancer, its microenvironment, and in communication between cells, serving as information carriers (Table 2). They can influence the development and progression of cancer by promoting the invasion, proliferation, and chemoresistance of cancer cells and metastasis [101]. On the other hand, there are lncRNAs with potential anticancer activity. Research on lncRNAs in HCC has great potential and can be used for faster diagnosis and the production of targeted drugs.

#### 2.3.3. Circular RNA

Recent studies suggest the involvement of circRNAs in the immune response [121]. Moreover, many studies have highlighted the role of these molecules, their functions in viruses and hosts, and interactions between them [122]. It is necessary to determine how viral circRNAs are regulated so that researchers can determine the role of these molecules in cancer pathogenesis [123]. Jost et al. [124] demonstrated that the in vitro artificial creation of circRNA sponges to sequester miR-122, a key molecule for HCV, led to the inhibition of viral protein production. Biomedical engineering research into the creation of modified circRNAs demonstrated the potential for regulating the expression of miR-122 [125]. This could be the basis for targeted pharmacotherapy.

circRNAs can contribute to the progression of HCC by modulating gene expression, apoptosis, and/or metastasis by interacting with miRNAs and altering the translation of specific proteins [126]. Furthermore, certain circRNAs could be potential biomarkers of HCC. Aborehab et al. [127] reported that circSERPINA3 is upregulated in the plasma of HCV and HCV-induced HCC groups, indicating a poor prognosis. In addition, sponging miR-944 leads to the downregulation of this molecule. Moreover, the area under the curve (AUC) values for circSERPINA3 and miR-944 in the HCV and the control group were 0.935 ± 0.0379 (95% confidence interval [CI] 0.8607–1.009, *p* < 0.001) and 0.982 ± 0.0147 (95% CI 0.9538−1.012, *p* < 0.001), and the AUC values for the HCC group and the control group were 0.982 ± 0.012 (95% CI 0.9588–1.007) and 0.981 ± 0.014 (95%CI 0.9527−1.010). Other promising biomarkers for HCC in patients infected with HCV are circSMARCA5 and SMARCA5mRNA [128]. The authors noted a statistically significant negative correlation between circSMARCA5 and SMARCA5mRNA and ALP, GGT, total bilirubin, and AFP. In addition, a receiver operating characteristic (ROC) curve analysis for cases diagnosed with low-stage HCC showed that the AUC for circSMARCA5 at a cut-off point of 4.55 yielded a specificity of 83.8% and a sensitivity of 91.7%. Perhaps combining this biomarker with classical biomarkers like AFP could improve the detection of HCC. miR-331-3p (AUC = 0.726, *p* < 0.001) may also have applications in the diagnosis of HCV-associated HCC [129]. This molecule is sponged by hsa_circ_0051443. In addition, this biomarker is significantly reduced in HCC cells (AUC = 0.8089, *p* < 0.001) [130]. As reported by Gosh et al. [131], HBV/HCV-related HCC involves the dysregulation of miR-10b, miR-21, and miR-182. Each of these miRNAs can be sponged circRNAs, and they may also have use in HCC diagnosis or therapy. The expression of hsa_circ_0070269 correlates negatively with the stage and size of HCC (*p* = 0.05); it sponges the upregulated miR182/NPTX1 axis and could be a potential therapeutic target [132]. Another potential biomarker is circ-102,166: it sequesters miR-182, and its expression level in HCC cells correlates positively with the tumour size (*p* = 0.03) and vessel invasion (*p* = 0.022) [133]. Although the above studies are inconclusive, they provide promising results. Future studies should focus on circRNAs and determine their potential as biomarkers or therapeutic targets.

## 3. HCV Infection and Immune Cells

The HCV virus causes chronic infection through many mechanisms that alter or impair the host’s immune response. One way is to block the expression of HLA class I. Mechanically, this is achieved by the HCV NS4A/B protein, which inhibits the movement of the endoplasmic reticulum (ER) to the Golgi apparatus. Consequently, MHC-I presentation decreases, and specific CD8+ T cells cannot recognise HCV-infected hepatocytes. This leads to a situation in which T lymphocytes will be produced in an impaired form because the immune system is not properly activated [134]. Additionally, the immune response of NK cells in people with chronic hepatitis C is also impaired. It has been proven that HCV patients have a reduced number of cytotoxicity receptors, such as NKp46 and NKp30, along with an increased number of inhibitory receptors, such as CD94/NKG2A. The pathological expression of NK receptors negatively affects the activation of dendritic cells (DC), which are crucial in the activation of T lymphocytes [135,136]. Tacke et al. demonstrated that the accumulation of CD33(+) myeloid-derived suppressor cells (MDSC) is increased in the presence of HCV. This leads to the ROS-mediated inhibition of T cell reactivity [137]. Impaired T cell response ultimately leads to the development of liver cirrhosis in the course of chronic HCV infection. Sreenarasimhaiah et al. showed differences in the responses of CD8 and CD4 T cells to HCV in patients with hepatitis and cirrhosis. The CD8 CTL response to five epitopes derived from the core proteins, NS5, NS4, and NS3, was examined. A lower CD8 CTL response was demonstrated in patients with cirrhosis compared to patients with inflammation [138]. As is known, CD8+ and CD4+ T lymphocytes are abundant in the environment of malignant tumours. Patients suffering from HCC with a large number of CD8+ T lymphocytes showed a better prognosis [139]. In summary, chronic HCV infection affecting immune cells, including T lymphocytes, may cause not only chronic infection but may also have a potential impact on the inability to limit the development of HCC in liver cirrhosis.

Barili et al. showed that HCV-specific CD8+ T cells at the beginning of infection are characterised by the upregulation of the transcription of genes related to DNA damage, the CD28-dependent PI3K/Akt anabolic pathway, the increase in p53 signalling, and the impairment of mitochondrial and glycolytic functions, among others. However, this regulation does not contribute to the proper antiviral response, but rather to its impairment. The author draws attention to histone methyltransferase inhibitors, which can reverse this effect by, for example, increasing the level of GLUT1, decreasing the level of PD-1 expression in CD8+ T cells, and increasing positive CD8+ T cells producing IFN-γ [140]. Chronic HCV infection induces oxidative stress, which, in turn, increases the expression of a class IIa histone deacetylase (HDAC), HDAC9. In addition, the increase in HDAC9 expression contributes to the increased transcription of rate-controlling gluconeogenic enzymes by the deacetylation of nuclear FoxO1, which is responsible for glucose metabolism in the liver. Mechanically, the deacetylation of nuclear FoxO1 increases binding to the FoxO1 DNA gluconeogenic gene promoter, leading to increased gluconeogenesis. These studies indicate that chronic HCV infection leads not only to fibrosis and steatosis but may also result in impaired glucose homeostasis and type 2 diabetes [141]. Furthermore, to activate gene transcription, the methylation of histone H3 lysine is necessary. The HCV virus contributes to the increase in the expression of Set7, which can activate the methylation of nonhistones such as DNMT1, STAT3, NF-κB, and p53, contributing to carcinogenesis. High Set7 expression also contributes to the inhibition of the translocation of IRF3 and IRF7 into the nucleus, which activate type I IFN production [142].

Hammad et al. reported that has-miR-155-5p and has-miR-21-5p were associated with the differentiation of subunits of monocytes. Intermediate monocytes (CD14+CD16+) are one of three subtypes of monocytes characterised by an enhanced expression of Il-12 and class II molecules. Furthermore, CD14+ monocytes secrete CCL2, CCL3, and IL-6 [143,144]. An increase in intermediate monocytes in the peripheral blood and the enhanced expression of plasma has-miR-155-5p and has-miR-21-5p were observed in patients with HCC compared to patients with liver cirrhosis, which suggests that they may serve as biomarkers of progression from liver cirrhosis to HCC [145]. In CD14+ monocytes from patients with chronic HCV, miR-146a expression was elevated. A study reported that the inhibition of miR-146a in CD14+ monocytes led to reductions in TGF-β1, IL-10, and IL-23 expression, which are responsible for T cell differentiation [146]. In the serum of patients with chronic HCV infection, increased levels of miR-155 and miR-122 were observed. Moreover, an elevated expression of miR-155 was observed in monocytes. Interestingly, in chronic HCV infection, the core protein, together with the NS2 and NS3 non-structured proteins, promote the expression of miR-155, which stimulates the production TNFα [73]. Moreover, the increased expression of miR-155 extends the half-time of TNFα mRNA [147]. Therefore, the virus can affect both pro- and anti-inflammatory responses. However, its interaction with pro-inflammatory mediators seems to be complex; it could depend on cellular context or on other regulatory mechanisms. The core protein downregulates has-miR146b-5p in T cells and monocytes, which is involved in the development of chronic HCV infection. The application of the has-miR146b-5p inhibitor in THP-1 cells resulted in reduced expressions of TNFα, INF-α, and IL-12. The 1(OH) VitaminD3 administration enhances the expression of miR146b-5p in monocytes, thus preventing persistent HCV infection development [148].

The pathological activation of neutrophiles and monocytes leads to the formation of myeloid-derived suppressor cells (MDSCs). Inflammation, infection, and cancer promote the expansion of these cells [149,150]. In patients with chronic HCV infection, a greater presence of MDSCs is observed. These cells stimulate the expression of the signal transducer and activator of transcription factor 3 (STAT-3) and regulatory T cell (Treg) development. It was observed that miR-124 expression in MDSCs was reduced. miR-124 downregulates the expression of STAT-3 and Treg Fox-p3+ together with IL-10. Notably, miR-124 overexpression decreases the expression of STAT-3 [151]. Another study demonstrated that supernatants from hepatocytes and exosomes derived from patients with HCV (HCV-Exo) can induce the differentiation of monocytic myeloid cells to MDSCs through the suppression of miR-124. Moreover, it was reported that chronic HCV infection leads to MDSC-mediated upregulation of T follicular regulatory (TFR) cells, resulting in an elevated expression of Il-10 and a reduced T follicular helper (TFH) cell fraction, which is associated with decreases in the INF-γ and IL-21 levels [152]. The presence of HCV contributes to the overexpression of miR-21. In HCV, three non-structural proteins, NS3, NS4A, and NS55, induce the c-Jun and c-Fos signalling pathway, resulting in the upregulated expression of miR-124. This molecule decreases the levels of INF type I, resulting in greater production of HCV in hepatocytes [153]. miR122 also enhanced the replication of HCV virus in liver cell lines [154]. HCV core protein downregulates the expression of miR-125b and upregulates IL-6, Il-10, and TNFα expression in THP-1 cells. The overexpression of cytokines is reversed by miR-125b mimic. This may suggest that miR-125b abrogates pro-inflammatory responses in HCV-infected cells [155]. miR-122 is necessary for HCV replication. It binds to two sites in the 5′Untrenstated region (UTR) of the internal ribosome entry sequence (IRES) [58,156]. A study by Li and colleagues reported that the overexpression of miR-130a reduced miR-122 expression and increased INFα and INFβ. These observations suggest that miR-130a is involved in the reduction in HCV replication and may serve as a potential drug target [157]. A study by Li et al. reported HCV infection in CD4+ T cells reduced the expression of miR-181a and increased DUSP6. The reconstruction of miR-181a, which regulates CD4+ T cell responses, improved T cells functionally through the DUSP6 pathway [158].

LncRNA BST2-2 (lncBST2-2) enhances the antiviral response by increasing the interaction of TBK1 and IRF3, binding to the DNA-binding domain of IRF3 and thereby enhancing INF production. The increased expression of lncBST2-2 has been demonstrated not only in HCV infection but also in vesicular stomatitis virus (VSV) or Newcastle disease virus (NDV) infection [159]. Another lncRNA, Linc-Pint, enhances the innate immune response. More specifically, Linc-Pint, by binding to DDX24, prevents the formation of the DDX24-RIP1 complex, which activates IRF7, increasing the expression of IFN-β and IFN-α in replicating HCV-infected hepatocytes [160]. Additionally, HOXA transcript antisense RNA myeloid-specific 1 (HOTAIRM1) derived from CD33+ myeloid cells of patients with HCV promotes suppressive functions and the differentiation of myeloid-derived suppressor cells (MDSCs) by, among others, increasing the expression of the signal activator and transducer of transcription 3 [161].

## 4. Conclusions and Future Perspectives

To conclude, epigenetic mechanisms are key regulators of gene expression, the dysregulation of which is associated with pathological conditions. Viruses are known for impairing epigenetic mechanisms, which are suggested to participate in the pathogenesis of infections or virus-induced cancer development [162,163]. In the context of HCV, altered epigenetics can facilitate viral infection and the progression to HCC and mediate the invasiveness of cancer. Knowledge about altered epigenetic mechanisms should allow for an introduction of novel treatment methods. For instance, the identification of hypermethylated genes that make a cell permissive for HCV could suggest an introduction of demethylating therapy. Furthermore, the infection was found to increase epigenetic scars that seem to persist after antiviral treatment. Recently, chronic infection was found to alter chromatin accessibility, which will also not return to the normal state after treatment. An impaired chromatin condition was suggested to be associated with the pro-inflammatory behaviour of Treg cells, which will also persist after successful treatment [164]. Hypothetically, the use of histone-modifying agents could remove epigenetic scars to change the phenotype and inflammatory responses of immune cells. Importantly, ncRNAs play a crucial role in regulating major cellular processes. HCV uses miR-122 to enhance its replication, and thus, miravirsen was developed to suppress miR-122 activity. As the drug requires continuous research, agents that modulate the expression of ncRNAs could modulate invasive viral behaviour together with host immune responses. Future analyses of epigenetics in HCV infection are greatly needed, as epigenetic mechanisms play an important role in the pathogenesis of HCC as well.

Specifically, a recent study demonstrated differences in the methylation profile between HCV-associated fibrosis and HCC liver tissue [165]. Furthermore, knowing that the expression of miRNAs is altered in HCV infection, that the virus can modulate the expression of these molecules, and that miRNAs play a role in the pathogenesis of HCC, it should be mentioned that miRNAs are also suggested to contribute to the transformation of cirrhotic liver tissues to HCC. Interestingly, miRNA-based prophylactic treatment was recently demonstrated to significantly reduce tumour growth in vivo [166]. The identification of molecules that regulate HCV infection, the progression of fibrosis and cirrhosis to HCC, and HCC development could perhaps lead to a more rapid introduction of novel treatment strategies.

## Figures and Tables

**Table 1:** A summary of the microRNAs (miRNAs) involved in the pathogenesis of hepatitis C virus (HCV) infection and progression to hepatocellular carcinoma.

microRNA	Expression	Mechanism Linking to HCV Infection	Association with HCV-Related Hepatocellular Carcinoma	References
miRNA-122	elevated	Leads to increased translation and stabilisation of HCV RNA	Decreased miR-122 levels may be associated with poor prognosis	[59,64]
miRNA-122	decreased	Decreases significantly with the progression of fibrosis severity	Decreased miR-122 levels may be associated with liver cancer metastasis	[62,65]
miRNA-155	increased in both serum and PMBC	Affects replication and life cycle of virus	Mediates proliferation and carcinogenesis due to increased Wnt signalling	[70,71,72]
miRNA-223	elevated in plasma, decreased in liver cells	Long-term immune response	Affects fibrosis through NOD receptors	[75,76,78]
miRNA-29	elevated in serum, decreased in liver cells	Suppression of HCV replication	Suppressive role	[82,83,87]
miRNA-34	elevated in serum	Cell cycle regulation	Suppressive role	[89,91,92]

**Table 2:** A summary of long non-coding RNAs (lnRNAs) involved in the pathogenesis of hepatitis C virus (HCV) infection and progression to hepatocellular carcinoma.

LncRNA	Functions	Mechanism	References
GAS5	Inhibiting HCV replication	Blocks NS3	[95]
UCA1	Antiviral response	Affects miR-145-5p/SOCS7/INF pathway	[96]
EGOT	Antiviral response	Reduces IFN	[97]
HOTAIR	Viral release	Affects lipid metabolism	[95]
IGF2-AS	Viral release	Increases PI4P	[95]
7SK	Viral release	Increases PI4P	[95]
IFI6	Increasing HCV replication	Modifies histones	[101]
Linc-Pint	Inhibiting HCV replication	Binds to serine-arginine protein kinase 2 (SRPK2)	[101]
NEAT1	HCC oncogene	Increases BGH3 concentration	[102]
lncTCF7	Tumour propagation	Activates Wnt signalling	[104]
LINC00624	Removing inhibitory effect on transcription in HCC	Disrupts formation of HDAC6-TRIM28-ZNF354C transcriptional corepressor complex	[105]
HULC	Promoting HCC cells growth and autophagy	Stimulates cyclin D1 and inhibits P21 WAF1/CIP1 in human liver cancer stem cells Manipulates pool of lipids HULC transcription may be regulated by HCV core protein via RXRA	[107,108]
MEG3	Tumour suppressor	Upregulation of tumour suppressors miRNA152 and p53 and downregulation of oncogenes TGF-b, BCL-2, and DNMT1 Induces apoptosis in HCC cell line Regulates expression of miRNA-10a-5p and PTEN	[110,111]
CASC2	Tumour suppressor	Inhibits MAPK pathway	[112]
LINC01189	Reducing 5-FU chemoresistance and inhibiting HCC proliferation	Inhibits hsa-miR-155-5p	[109]
HEIH	Biomarker	HCV-related HCC, gastric cancer, cirrhosis	[114,115,116]
TUG1	Biomarker	HCV-related HCC, gastric cancer, osteosarcoma, urothelial carcinoma of the bladder, esophageal squamous cell carcinoma	[113]
MALAT1	Biomarker	HCV-related HCC, breast cancer, lung cancer	[118]
LINC01419	Biomarker	HCV- and HBV-related HCC	[120]

## Data Availability

Not applicable.
